# An explorative study on the interaction of cyclin-dependent kinase inhibitor 2B antisense RNA 1 (*CDKN2B-AS1/ANRIL*) gene polymorphism with obesity on periodontitis among Norwegian adults

**DOI:** 10.2340/aos.v84.44368

**Published:** 2025-08-14

**Authors:** Natalia Petrenya, Laila A. Hopstock, Elin Hadler-Olsen, Farah Asa’ad, Lena Larsson, Svetlana N. Zykova, Gro Eirin Holde, Alexandre R Vieira, Birgitta Jönsson

**Affiliations:** a The Public Dental Health Service Competence Centre of Northern Norway, Tromsø, Norway; b Department of Health and Care Sciences, UiT The Arctic University of Norway, Tromsø, Norway; c Department of Medical Biology, Faculty of Health Sciences, UiT The Arctic University of Norway, Tromsø, Norway; d Department of Oral Biochemistry, Institute of Odontology, The Sahlgrenska Academy, University of Gothenburg, Gothenburg, Sweden; e Center for Research and Education, University Hospital of North Norway, Tromsø, Norway; f Hormone Laboratory, Department of Medical Biochemistry, Biochemical Endocrinology and Metabolism Research Group, Oslo University Hospital, Oslo, Norway; g Department of Clinical Dentistry, Faculty of Health Sciences, UiT The Arctic University of Norway, Tromsø, Norway; h School of Dental Medicine, East Carolina University, Greenville, NC, USA; i Department of Periodontology, Institute of Odontology, The Sahlgrenska Academy, University of Gothenburg, Gothenburg, Sweden

**Keywords:** periodontitis, obesity, *ANRIL*long non-coding RNA, single nucleotide polymorphism, genetic variation

## Abstract

**Objective:**

*ANRIL* is a pleiotropic gene with a strong link to periodontitis. *ANRIL* gene variant rs1537373 is associated with altered *CDKN2B* gene expression, which is linked to obesity. In this explorative, cross-sectional population-based study, we aimed to investigate the hypothesis that *ANRIL* (rs1537373) T > G may be associated with periodontitis through interactions, focusing on rs1537373×obesity interaction.

**Methods:**

Genotyping for *ANRIL* (rs1537373), and clinical and periodontal examination were performed in 3,554 participants (aged 40–93 years, 52% women) from the seventh survey of the Tromsø Study (2015–2016), Norway. We defined periodontitis stage based on radiographic bone loss (2018 American Academy of Periodontology and the European Federation of Periodontology classification).

**Results:**

The individual association between rs1537373 and periodontitis, as well as multiplicative and additive interactions between rs1537373 and age, sex, smoking, and obesity on periodontitis under a recessive model were studied. We found multiplicative and additive interactions between rs1537373 and obesity. When compared with G (guanine) T (thymine)/TT genotype and no obesity, GG genotype and obesity was associated with higher odds for periodontitis stage III–IV (odds ratio (OR) = 2.47, 95% confidence interval [CI] = 1.48–4.12, *p* = 0.001; relative excess risk due to interaction = 1.51, 95% CI = 0.26–2.77, *p* = 0.018; attributable proportion due to interaction = 0.61, 95% CI = 0.35–0.87, *p* < 0.001).

**Conclusion:**

Our findings suggest that rs1537373 GG genotype and obesity were jointly associated with periodontitis stage III–IV in this Norwegian population.

## Introduction

Periodontitis is an inflammatory disease associated with the formation of dysbiotic biofilms and characterised by the progressive destruction of tooth-supporting tissues that can lead to tooth loss and impaired mastication [1]. Multiple studies have demonstrated a link between obesity and periodontitis [2]; however, the mechanisms that explain the links between these conditions are not completely understood. The association between obesity and periodontitis may be mediated through interrelated etiological pathways [3], including microbiome changes [4]. Obesity can promote pro-inflammatory responses by enhancing T helper 1 cell activity and M1-polarised macrophages, while decreasing T helper 2 cell responses [5, 6]. This may lead to a chronic inflammatory state, insulin resistance [7], and oxidative stress [8]. Myeloid-derived suppressor cells are significantly expanded during obesity and exhibit potent immunosuppressive functions. During obesity, this cell population demonstrates enhanced osteoclastogenic capacity, contributing to greater alveolar bone loss in experimental periodontitis [9]. There is limited knowledge about the genetic link between obesity and periodontitis; only a few epidemiologic studies have focused on this issue [10–12].

Genetic locus cyclin-dependent kinase inhibitor 2B antisense 1 (*CDKN2B-AS1*) RNA, known as *ANRIL* (chromosome 9, p21.3), has consistently been reported in genome-wide association studies (GWAS) to be associated with periodontitis [13–17]. Notably, specific polymorphisms located in the *ANRIL* locus, such as rs1333048, rs1333042, rs2891168, and rs496892, have been repeatedly implicated in periodontitis in Whites [13], with the first three of these polymorphisms located in a linkage disequilibrium block [18]. It must be emphasised that *ANRIL* is a pleotropic gene and the most robust marker for coronary artery disease (CAD) [16]. *ANRIL* polymorphism in intron 3 rs1537373 has been previously linked to three comorbidities of periodontitis [19–21], that is lipid levels [22, 23], artery calcification [24], and pancreatic cancer [25]. The G allele of this *ANRIL* variant was found to be a risk allele [24, 25], and this risk allele represented higher enhancer activity [25]. rs1537373 is in the strong linkage disequilibrium with periodontitis markers rs1333048, rs1333042, and rs2891168, as well as CAD marker rs1333049. Therefore, rs1537373 may be considered as a proxy single nuclear polymorphism (SNP) tagging the periodontitis and CAD risk haplotype.

Previous studies have suggested the potential mechanisms, including polymorphism and epigenetic change, by which the *ANRIL* regulated obesity. Results from a recent experimental study (chr4 Δ70kb/70kb mice) supported its involvement in bone and fat tissue metabolism [26]. *ANRIL* has been shown to regulate its neighbour tumour suppressor genes cyclin-dependent kinase inhibitor 2A (*CDKN2A)* and 2B (*CDKN2B)* [27]. Several studies have found that the *ANRIL* gene variant rs1537373 can influence enhancer activity and cause altered *CDKN2B* gene expression [25, 28, 29]. *CDKN2B* gene is important for adipogenesis. It has been demonstrated that knock-down of *CDKN2B* expression in a mouse adipocyte cell line results in increased adipogenesis [30]. Svensson et al. showed that the *CDKN2B* gene is highly expressed in subcutaneous adipose tissue, its expression is linked to markers of hepatic steatosis and postprandial triacylglycerol clearance, and the polymorphism in *ANRIL* modifies *CDKN2B* expression in a body mass index (BMI)-dependent fashion; therefore, it has been suggested that the *ANRIL* polymorphism may be involved in ectopic fat accumulation [31]. *CDKN2A* was found to be involved in the balance between adipogenesis and senescence, and plays a role in adipocyte insulin sensitivity and lipid storage, inflammation, as well as oxidative activity and browning [32]. Moreover, methylation of the *ANRIL* promoter has been related to adiposity [33], as well as decreased bone size, mineral content, and mineral density [34] in children. In addition, *ANRIL* has been implicated in regulation of genes involved in glucose and fatty acid metabolism [14], glycolipid metabolism [35], and type 2 diabetes mellitus (T2DM) [36].

Assessment of the interaction between genotype and environment (G×E) is important in the understanding of the pathogenesis of multifactorial diseases such as periodontitis. Evaluating (G×E) interaction under an additive risk model (i.e. additive interaction) has gained attention because additive interactions might shed light on biological mechanisms and can reveal the benefits of targeted interventions based on genetic susceptibility [37]. Given that rs1537373 has previously not been identified as a risk polymorphism for periodontitis but is linked to altered *CDKN2B* gene expression, which is associated with obesity, we hypothesised that obesity may modify associations between rs1537373 and periodontitis. Therefore, our explorative study aimed to test the hypothesis that *ANRIL* (rs1537373) T>G may be associated with periodontitis through interactions, focusing on rs1537373×obesity interaction.

## Materials and methods

### Study design and study participants

**Figure 1:**
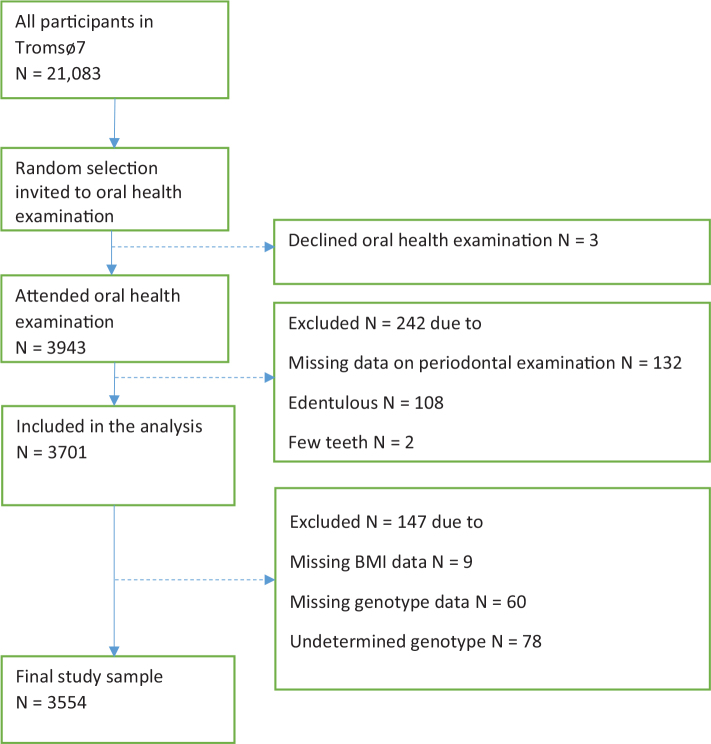
Flow chart of the present cross-sectional study. BMI: body mass index.

All inhabitants aged ≥40 years residing in the Tromsø municipality, Norway, were invited to the Tromsø7, 2015–2016, and 65% attended (*N* = 21,083, aged 40–99 years, 53% women). Study design and data collection details were described elsewhere [38]. Data collection included questionnaires, interviews, clinical examinations, and biological sampling (i.e. blood and saliva). A random subsample of 3,943 Tromsø7 participants also attended a dental examination. Status of *ANRIL* genotype, obesity, and periodontitis were available for 3,554 participants 40–93 years of age (52% women) (Figure 1).

### Dental examination and case definition of periodontitis

Full-mouth periodontal assessments were performed at four sites per tooth for all natural teeth, excluding third molars. The interproximal radiographic marginal bone level (RBL) was measured at two sites per tooth on panoramic radiographs as described by Holde et al. [39]. The clinical examination was performed at the same time as the panoramic radiograph was taken. Calibration of radiographic bone level measurements was done on two occasions with two sets of orthopantomographs for each assessor, where the assessors (*n* = 3) were compared to both themselves and an experienced dentist (GEH). The median of intra-class correlation coefficient (ICC) for interrater agreement was 0.76 (range 0.68–0.77) for the first calibration and 0.76 (range 0.60–0.93) 3 months later. The median ICC for interrater agreement was 0.69 (range 0.53–0.76) for the first calibration and 0.75 (range 0.65–0.84) for the second calibration.

Periodontitis stages were classified according to the classification of periodontal and peri-implant diseases by the American Academy of Periodontology and the European Federation of Periodontology (AAP/EFP) [40, 41]. Participants were defined as a periodontitis case if interdental RBL was detectable at ≥2 non-adjacent teeth. Periodontitis cases were further defined as stage I (<15% RBL), stage II (15%–33% RBL), and stage III–IV (>33% RBL). To avoid shifting of stage resulting in participants with different levels of bone loss being included in the same stage (e.g. participant with stage II bone loss and pocket depth [PD] 6 mm shifting to stage III), the complexity factor periodontal PD was not considered. Stage III and IV were not differentiated due to a lack of data on the reasons for tooth loss, furcation involvement, and tooth mobility. In the analysis, we combined participants classified as no periodontitis with stage I.

### Polymorphism selection, isolation of DNA, and genotyping

#### Polymorphism selection

All participants who took part in a dental examination in Tromsø7 were tested for selected SNPs in some pre-specified genetic regions (not the entire genome) related to inflammation, epigenetic function [42], and periodontal and dental tissues. The rs1537373 polymorphism was the only polymorphism in the *ANRIL* gene that has been assessed. The selection of SNPs combined three criteria: the frequency of the less common allele predicted to be higher than 40%, the location of the variant within the gene, and the existence of an optimised assay for TaqMan chemistry.

#### Contextualisation of the chosen single nuclear polymorphism in the present study

rs1537373 is located in a highly pleiotropic gene, *ANRIL*, which is robustly associated with periodontitis [13, 15, 18]. The functional understanding of the *CDKN2A/B* locus remains incomplete [43]. rs1537373 has been shown to be located within an enhancer region, with rs1537373-G allele associated with higher enhancer activity. It has been suggested that rs1537373 may be a functional variant because of its ability to induce differential binding of transcription factors, thereby regulating the expression of genes such as *CDKN2B* [25, 28, 29]. The *CDKN2B* gene encodes the tumour suppressor protein p15INK4b, which regulates the cell cycle by inhibiting CDK4 and CDK6. Dysregulation of *CDKN2B* has been implicated in metabolic disorders, including obesity [30, 31], as well as cardiovascular diseases (CVDs) and various cancers. Moreover, rs1537373 has been linked to altered lipid levels [22, 23], excessive calcium deposits/artery calcification [24], and pancreatic cancer [25], outcomes that were reported to comorbid with both periodontitis [19–21] and obesity [44–46]. In addition, rs1537373 is in strong linkage disequilibrium with several *ANRIL* SNPs, which have been repeatedly associated with periodontitis; thus, it may be considered a proxy SNP tagging the periodontitis risk haplotype.

#### Isolation of DNA

Unstimulated saliva samples (up to 2 mL) collected during the dental examination were stored at room temperature in the Oragene DNA Self-Collection System (DNA Genotek Inc., Murrieta, CA, USA) until DNA extraction, which was performed within the recommended timeframe to ensure high-quality DNA. DNA was isolated using Agencourt DNAdvance kit (Beckman Coulter Genomics) following the manufacturer’s instructions. Saliva samples were incubated at 50°C for 1 hour. Then, 500 μL of the sample were transferred into a new 1.5 mL microcentrifuge tube, and 200 μL of the Bind1 binding buffer was added, followed by 340 μL of the Bind2 buffer containing magnetic DNA-binding beads. The mixture was mixed thoroughly and incubated at room temperature for 1 minute. A magnet was applied for 8 minutes to allow separation, after which the supernatant was aspirated and discarded. The magnet was removed, and 700 μL of 70% ethanol was added to resuspend the magnetic beads from the bottom of the tube. Upon completion of two washing steps with 70% ethanol, the DNA was eluted using 50 μL of elution buffer. Subsequently, 40 μL 40 μL of supernatant was transferred to a clean tube and stored at -80°C upon re-application of the magnet. DNA concentrations were assessed by measuring the absorbance at 260 nm using a spectrophotometer.

#### Genotyping

For downstream analyses, the DNA extracts were diluted to 2 ng DNA/μL in TE buffer (10 mM Tris-HCl, 1 mM EDTA, pH 8.0). Genotyping was then performed using TaqMan-based polymerase chain reaction (PCR) assay. All reagents, including primers, probes, and master mix were purchased from Applied Biosystems (Foster City, CA, USA), and amplification products were detected and quantified using the Applied Biosystems 7900 HT Sequence detection system. For the allelic discrimination assay, two TaqMan probes, TaqMan PCR master mix, and SNP assay were used (Applied Biosystems, Foster City, CA, USA). The thermal cycle was as follows: a start with a hold cycle at 95°C for 10 minutes, followed by 40 amplification cycles at 92°C for 15 seconds and at 60°C for 1 minute.

### Covariates

Height and weight were measured with a Jenix DS-102 automatic electronic stadiometer (DongSahn Jenix, Seoul, Korea). Body mass index was calculated as weight in kilograms divided by the square of height in meters (kg/m^2^) and categorised as no obesity (<30 kg/m^2^) or obesity (≥30.0 kg/m^2^).

Information about (1) education (primary/partly secondary (up to 10 years of schooling), upper secondary (minimum of 3 years), or college/university education); (2) smoking status (never, former, or current); (3) leisure-time physical activity by the Saltin and Grimby questionnaire [47] (sedentary, low, or moderate/vigorous); (4) toothbrushing frequency (< twice a day or ≥ twice a day); (5) use of antihypertensives (current use by answering to ‘Do you use antihypertensive medication?’ and/or use of antihypertensives C02, C03, C07, C08, and/or C09 from a written list of brand names of regularly used medications coded by the anatomic therapeutic and chemical [ATC] classification system); (6) use of antidiabetics (tablets and/or insulin); (7) diabetes (never, previous, or current); and (8) family history of premature coronary heart disease (CHD) (myocardial infarction before the age of 60 years in ≥ 1 first-degree family member); (9) information on the CVD: heart attack, cerebral stroke/brain haemorrhage, angina pectoris (heart cramp), and/or cardiac surgery was taken from Tromsø7 self-administered questionnaires. Non-fasting venous blood samples were collected with standard methods and analysed at the Department of Laboratory Medicine, University Hospital of North Norway (ISO certificate NS-EN ISO 1-5189), for serum total cholesterol, low-density lipoprotein cholesterol (LDL-C), high-density lipoprotein cholesterol (HDL-C), and triglycerides by enzymatic colorimetric methods with a Cobas 8000 c702 (Roche Diagnostics, Mannheim, Germany); high-sensitivity C-reactive protein (hs-CRP) by a particle-enhanced immunoturbidimetric assay (Cobas 8000, Roche diagnostics, Mannheim, Germany); and glycated haemoglobin (HbA1c) by high-performance liquid chromatography (Tosoh G8:Tosoh Bioscience, San Francisco, USA). High LDL-C was defined as LDL-C ≥ 3.4 mmol/L. Low HDL-C was defined as HDL-C < 1.0 mmol/L in men and < 1.3 mmol/L in women. High triglycerides were defined as triglycerides ≥ 1.7 mg/dL. Diabetes was defined by self-reported current diabetes and/or current use of diabetes medications and/or HbA1c ≥ 6.5%.

### Statistical analysis

The differences in characteristics according to GT/TT and GG and genotype groups were evaluated by analysis of variance (ANOVA), Mann-Whitney U test, Poisson regression, and Pearson’s χ^2^ test in agreement with the type of variable. We calculated minor allele frequency, and the Hardy–Weinberg equilibrium (HWE) test was used to determine whether the periodontal groups in the total sample, and in the subsamples stratified by obesity, were a random group of the target population. The Akaike Information Criterion (AIC) and the Bayesian Information Criterion (BIC) were used for optimal model selection; the recessive model was chosen. Interactions between *ANRIL* genotype and unmodifiable (age and sex) and modifiable (smoking and obesity) risk factors were analysed by multiplicative and additive scales. Additive interaction effect was evaluated by relative excess risk due to interaction (RERI) and attributable proportion due to interaction (AP), their 95% confidence intervals (CIs), and *p*-value to determine statistical significance [48, 49]. While RERI is the part of the total effect that is due to interaction, AP is the proportion of the combined effect that is due to interaction. Their calculation formulas are: RERI = HR11-HR01-HR10+1 and AP = RERI/HR11. Since no interaction between genotype and age, sex, and smoking on periodontitis was observed, these data were not presented. The selection of covariates for models was based on univariant tests and stepwise selection. The final model testing associations between *ANRIL* genotype, obesity, and periodontitis were adjusted for age, sex, education, smoking, toothbrushing frequency, low HDL-C, and hs-CRP.

We also present unadjusted and adjusted regression models between *ANRIL* genotype and obesity as outcome under a recessive model in the total sample, and stratified by periodontitis subgroup. Results from multiple binomial logistic regression models are presented as adjusted ORs and 95% CIs. All tests were two-tailed, with *p*-values <0.05 considered statistically significant.

Latent class analysis (LCA) was performed subsequently because we wanted to confirm our findings by using an alternative statistical approach and to address methodological challenges that arise in subgroup analysis, including Type I error and low statistical power. The description of LCA can be found in Supplementary Material online.

All statistical analyses were performed in STATA software (STATA, StataCorp, College Station, TX, USA, version 17.0).

## Results

### Population characteristics

**Figure 2:**
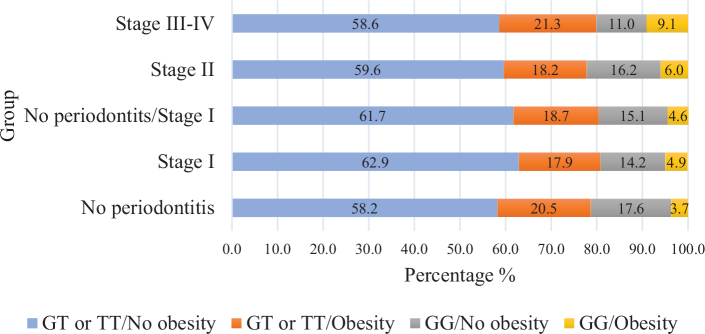
Percentage of participants according to *ANRIL* genotype and obesity within each periodontitis group.

In the study population, 25% were obese, 45% had periodontitis stage II, and 13% had periodontitis stage III–IV (Table 1). The GT/TT and GG groups were similar, except that the proportion of participants with obesity (*p* = 0.019), family history of CHD (*p* = 0.011), and elevated triglycerides (*p* = 0.007) was higher in the GG than the GT/TT group (Table 1). Among participants with obesity, 21% in the GG genotype group had periodontitis stage III–IV compared to 15% in the GT/TT group (*p* = 0.041) (Table 1). In total, 5% in the no periodontitis/stage I group compared to 9% in the stage III–IV group had both the GG genotype and obesity (Figure 2).

**Table 1:** Population characteristics of the total study sample according to *ANRIL* gene variant (*n* = 3,554).

Characteristics	Total	ANRIL genotype		
	*n* = 3,554	GT/TT *n* = 2,813 (79.2%)	GG *n* = 741 (20.8%)	*p* ^j^
Age (years), mean (SD)	57.6 (11.1)	57.7 (11.0)	56.9 (11.2)	0.066^k^
Age group (years), *n* (%)				
40–49	1,019 (28.7)	787 (28.0)	232 (31.3)	
50–59	1,023 (28.8)	807 (28.7)	216 (29.1)	
60–69	949 (26.7)	774 (27.5)	175 (23.6)	
≥ 70	563 (15.8)	445 (15.8)	118 (15.9)	0.130
Sex, *n* (%)				
Women	1,833 (51.6)	1,429 (50.8)	404 (54.5)	0.071
Education, *n* (%)				
Primary/partly secondary	819 (23.4)	660 (23.8)	159 (21.9)	
Upper secondary	1,044 (29.9)	826 (29.8)	218 (30.0)	
College/university	1,632 (46.7)	1,283 (46.3)	349 (48.1)	0.523
Smoking status, *n* (%)				
Never	1,500 (43.0)	1,190 (43.1)	310 (42.8)	
Former	1,520 (43.6)	1,204 (43.6)	316 (43.6)	
Current	466 (13.4)	368 (13.3)	98 (13.5)	0.985
Physical activity, *n* (%)				
Sedentary	476 (13.8)	373 (13.7)	103 (14.4)	
Light	2,003 (58.2)	1,581 (58.0)	422 (58.9)	
Moderate/vigorous	962 (28.0)	770 (28.3)	192 (26.8)	0.704
Family history of CHD^a^, *n* (%)	834 (23.5)	634 (22.5)	200 (27.0)	**0.011**
Obesity^b^, *n* (%)	875 (24.6)	668 (23.7)	207 (27.9)	**0.019**
High LDL-C^c^, *n* (%)	422 (57.0)	1,645 (58.5)	422 (57.0)	0.453
Low HDL-C^d^, *n* (%)	481 (13.6)	375 (13.4)	106 (14.4)	0.463
Elevated triglycerides^e^, *n* (%)	1,032 (29.0)	787 (28.0)	245 (33.1)	**0.007**
Hs-CRP (mg/L), median (25th–75th percentile)	1.0 (0.5–2.1)	1.0 (0.5–2.1)	1.0 (0.5–2.0)	0.367^l^
HbA1c (%), mean (SD)	5.7 (0.7)	5.7 (0.7)	5.7 (0.6)	0.705^k^
Diabetes^f^, *n* (%)	250 (7.0)	200 (7.1)	50 (6.7)	0.730
CVD^g^, *n* (%)	347 (9.8)	272 (9.7)	75 (10.1)	0.715
Use of antihypertensives^h^, *n* (%)	896 (25.2)	705 (25.1)	191 (25.8)	0.691
Toothbrushing frequency^i^, *n* (%)	697 (20.0)	559 (20.3)	138 (19.0)	0.446
Number of teeth, mean (SD)	24.1 (5.4)	24.0 (5.4)	24.1 (5.4)	0.557^m^
Number of participants with ≥1 tooth with PD≥6mm, *n* (%)	485 (13.6)	382 (13.6)	103 (13.9)	0.821
Periodontitis groups, *n* (%)				
No periodontitis	380 (10.7)	299 (10.6)	81 (10.9)	
Stage I	1,098 (30.9)	888 (31.6)	210 (28.3)	
Stage II	1,602 (45.1)	1,247 (44.3)	355 (47.9)	
Stage III–IV	474 (13.3)	379 (13.5)	95 (12.8)	0.275
Periodontitis groups used in the analysis stratified by obesity subgroup				
No obesity				
Periodontitis groups, *n* (%)				
No periodontitis/stage I	1,135 (42.4)	912 (42.5)	223 (41.8)	
Stage II	1,214 (45.3)	955 (44.5)	259 (48.5)	
Stage III–IV	330 (12.3)	278 (13.0)	52 (9.7)	0.076
Obesity				
Periodontitis groups, *n* (%)				
No periodontitis/stage I	344 (39.3)	276 (41.3)	68 (32.9)	
Stage II	388 (44.3)	292 (43.7)	96 (46.4)	
Stage III–IV	143 (16.3)	100 (15.0)	43 (20.8)	0.041

BMI: body mass index; CHD: coronary heart disease; LDL-C: low-density lipoprotein-cholesterol; HDL-C: high-density lipoprotein cholesterol; CVD: cardiovascular disease; hs-CRP: high-sensitivity C-reactive protein; HbA1c: glycated hemoglobin. Bold values denote statistical significance at the *p* < 0.05 level.

a ≥1 first degree family member having suffered an acute myocardial infarction before the age of 60 years.x

b BMI ≥30 kg/m^2^;

c ≥3.4 mmol/l;

d Men <1.0 mmol/l; women <1.3 mmol/l;

e ≥1.7 mmol/l;

f Self-reported current diabetes and/or current use of diabetes medication and/or HbA1c ≥6.5%;

g Heart attack, cerebral stroke/brain haemorrhage, angina pectoris, and/or cardiac surgery;

h Current use by answering yes to the question ‘Do you use antihypertensive medication?’ and/or use of antihypertensives ATC C02, C03, C07, C08, and/or C09 from a written list of brand names of regularly used medications coded by the anatomic therapeutic and chemical (ATC) classification system;

i < twice a day;

j χ^2^ test unless otherwise indicated;

k Analysis of variance;

l Mann-Whitney U-test;

m Poisson regression.

The minor allele frequency G was 46% in the total sample and 45%, 46%, and 44% in the no periodontitis/stage I, stage II, and stage III–IV groups, respectively (Table 2). No departure from HWE was detected (Table 2). The absolute numbers of participants in the subgroups can be found in Table 2.

**Table 2:** Minor allele frequency, genotypes frequency, and HWE according to periodontitis in the total study sample and stratified by obesity subgroup (*n* = 3,554).

Allele and genotype categories	Total	No periodontitis/stage I	Stage II	Stage III–IV
Total study sample, *n* (%)	3,554 (100.0)	1,479 (41.6)	1,602 (45.1)	473 (13.3)
Allele frequency G, *n* (%)	3,241 (0.46)	1,344 (0.45)	1,484 (0.46)	413 (0.44)
Allele frequency T, *n* (%)	3,867 (0.54)	1,614 (0.55)	1,720 (0.54)	533 (0.56)
Genotype frequency, *n* (%)				
TT	1,054 (29.7)	426 (28.8)	473 (29.5)	155 (32.8)
GT	1,759 (49.5)	762 (51.5)	774 (48.3)	223 (47.1)
GG	741 (20.8)	291 (19.7)	355 (22.2)	95 (20.1)
HWE, *p*-value	0.887	0.133	0.255	0.365
Stratified by obesity subgroup				
No obesity, *n* (%)	2,679 (100.0)	1,135 (42.4)	1,214 (45.3)	330 (12.3)
Allele frequency G, *n* (%)	2,402 (0.45)	1,033 (0.45)	1,103(0.45)	266 (0.40)
Allele frequency T, *n* (%)	2,956 (0.55)	1,237 (0.55)	1,325 (0.55)	394 (0.60)
Genotype frequency, *n* (%)				
TT	811 (30.3)	325 (28.6)	370 (30.5)	116 (35.2)
GT	1,334 (49.8)	587 (51.7)	585 (48.2)	162 (49.1)
GG	534 (19.9)	223 (19.6)	259 (21.3)	52 (15.8)
HWE, *p*-value	0.731	0.149	0.327	0.714
Obesity, *n* (%)	875 (100.0)	344 (39.3)	388 (44.3)	143 (16.3)
Allele frequency G, *n* (%)	839 (0.48)	311 (0.45)	381 (0.49)	147 (0.51)
Allele frequency T, *n* (%)	911 (0.52)	377 (0.55)	395 (0.51)	139 (0.49)
Genotype frequency, *n* (%)				
TT	243 (27.8)	101 (29.4)	103 (26.5)	39 (27.3)
GT	425 (48.6)	175 (50.9)	189 (48.7)	61 (42.7)
GG	207 (23.7)	68 (19.8)	96 (24.7)	43 (30.1)
HWE, *p*-value	0.426	0.618	0.616	0.080

HWE: Hardy–Weinberg equilibrium

### Individual effect of rs1537373-G and obesity on periodontitis

rs1537373 polymorphism was not significantly associated with periodontitis in the model evaluating individual associations under the recessive model, after adjustment for age, sex, education, smoking, toothbrushing frequency, low HDL-C, and hs-CRP. However, obesity was associated with periodontitis stage III–IV compared with no periodontitis/stage I (odds ratio [OR] = 1.39, 95% confidence interval [CI] = 1.03–1.88, *p* = 0.030) (Table 3, Model 3).

**Table 3:** Individual associations of *ANRIL* genotype and obesity with periodontitis under recessive model.

Groups	Model 1^a^		Model 2^b^		Model 3^c^	
	OR (95% CI)	*p*	OR (95% CI)	*p*	OR (95% CI)	*p*
Stage II vs. no periodontitis/stage I						
Genotype						
GT/TT	1.00		1.00		1.00	
GG	1.16 (0.98, 1.38)	0.093	1.21 (1.00, 1.47)	0.077	1.20 (0.99, 1.45)	0.065
Obesity						
No obesity	1.00		1.00		1.00	
Obesity	1.05 (0.89, 1.24)	0.553	0.98 (0.80, 1.20)	0.866	0.98 (0.81, 1.19)	0.857
Stage III–IV vs. no periodontitis/stage I						
Genotype						
GT/TT	1.00		1.00		1.00	
GG	1.01 (0.78, 1.31)	0.951	1.17 (0.85, 1.61)	0.331	1.15 (0.84, 1.58)	0.386
Obesity						
No obesity	1.00		1.00		1.00	
Obesity	**1.43 (1.13, 1.80)**	**0.002**	**1.40 (1.03, 1.91)**	**0.032**	**1.39 (1.03, 1.88)**	**0.030**

OR: odds ratio; CI: confidence interval; HDL-C: high-density lipoprotein cholesterol; hs-CRP: high-sensitivity C-reactive protein. Bold values denote statistical significance at the *p* < 0.05 level.

a Model 1: Binominal logistic regression model included *ANRIL* genotype and obesity as independent variables;

b Model 2: Binominal logistic regression model included, in addition to *ANRIL* genotype and obesity, age, sex, education, smoking, physical activity, toothbrushing frequency, low HDL-C, hs-CRP, diabetes, and use of antihypertensives (selected based on significant univariate test);

c Model 3: Final binominal logistic regression model included, in addition to *ANRIL* genotype and obesity, age, sex, education, smoking, toothbrushing frequency, low HDL-C, and hs-CRP (selected based on stepwise selection, *p* < 0.2).

### Rs1537373-G and obesity combine effects to increase susceptibility to periodontitis

Table 4 presents the results of the interaction analysis, along with the absolute number of participants in each subgroup. A multiplicative interaction between obesity and the *ANRIL* genotype on periodontitis stage III–IV was detected (*p* = 0.005) (Table 4). We also observed a significant positive additive interaction. Obese participants with the GG genotype had higher odds for periodontitis stage III–IV compared with non-obese participants with the GT/TT genotype (OR = 2.47, 95% CI = 1.48–4.12, *p* = 0.001; RERI = 1.51, 95% CI = 0.26–2.77, *p* = 0.018; AP = 0.61, 95% CI = 0.35–0.87, *p* < 0.001).

**Table 4:** Multiplicative and additive interaction of *ANRIL* genotype and obesity on periodontitis under recessive model.

Groups	*N*	OR (95% CI), *p*	*n*	OR (95% CI), *p*	Multiplicative interaction^a^	Additive interaction measurements^b^	
					*p*	RERI OR (95%CI), *p*	AP OR (95%CI), *p*
Stage II vs. no periodontitis/stage I		GT/TT *n* = 2,435		GG *n* = 646			
No obesity	1,867	1.00	482	0.93 (0.75, 1.15), 0.494	0.238	0.35 (–0.17, 0.87), 0.192	0.26 (–0.06, 0.57), 0.117
Obesity	568	1.12 (0.90, 1.40), 0.314	164	1.36 (0.95, 1.94), 0.097			
Stage III–IV vs. no periodontitis/stage I		GT/TT *n* = 1,566		GG *n* = 386			
No obesity	1,190	1.00	275	0.84 (0.57, 1.24), 0.381	**0.005**	**1.51 (0.26, 2.77), 0.018**	**0.61 (0.35, 0.87), <0.001**
Obesity	376	1.12 (0.80, 1.58), 0.502	111	**2.47 (1.48, 4.12), 0.001**			

OR: odds ratio; CI: confidence interval; HDL-C: high-density lipoprotein cholesterol; hs-CRP: high-sensitivity C-reactive protein; RERI: relative excess risk due to interaction; AP: attributable proportion. Bold values denote statistical significance at the *p* < 0.05 level.

a Multiplicative interaction: binominal logistic regression model included *ANRIL* genotype, obesity, product term *ANRIL* genotype*obesity, age, sex, education, smoking, toothbrushing frequency, low HDL-C, and hs-CRP.

b Interaction on an additive scale means that the combined effect of two exposures is larger (or smaller) than the sum of the individual effects of the two exposures. ORs are adjusted for age, sex, education, smoking, toothbrushing frequency, low HDL-C, and hs-CRP. RERI and AP were calculated with the formulas: RERI = OR11 – OR10 – OR01 + 1, AP = RERI/OR11. The delta method introduced by Hosmer and Lemeshow was used to calculate the 95% CI of RERI and AP.

When stratified by obesity in the fully-adjusted final models (Table 5, Model 2), the GG genotype was associated with higher odds for both periodontitis stage II (OR = 1.50, 95% CI = 1.02–2.22, *p* = 0.042) and stage III–IV (OR = 2.14, 95% CI = 1.25–3.69, *p* = 0.006). Interaction between *ANRIL* genotype and age, sex, or smoking was not observed.

**Table 5:** Associations between *ANRIL* genotype and periodontitis under a recessive model stratified by obesity subgroup.

Groups	Stage II vs. no periodontitis/stage I				Stage III–IV vs. no periodontitis/stage I			
	Model 1^a^		Model 2^b^		Model 1^a^		Model 2^b^	
	OR (95% CI)	*P*	OR (95% CI)	*P*	OR (95% CI)	*p*	OR (95% CI)	*p*
No obesity								
Genotype								
GT/TT	1.00		1.00		1.00		1.00	
GG	1.11 (0.91, 1.36)	0.312	1.12 (0.90, 1.40)	0.316	0.76 (0.55, 1.06)	0.112	0.84 (0.56, 1.25)	0.395
Obesity								
Genotype								
GT/TT	1.00		1.00		1.00			
GG	1.33 (0.94, 1.90)	0.108	**1.50 (1.02, 2.22)**	**0.042**	**1.75 (1.12, 2.27)**	**0.014**	**2.14 (1.25, 3.69)**	**0.006**

OR: odds ratio; CI: confidence interval; HDL-C: high-density lipoprotein cholesterol; hs-CRP: high-sensitivity C-reactive protein. Bold values denote statistical significance at the *p* < 0.05 level.

a Model 1: Unadjusted binominal logistic regression model between *ANRIL* genotype and periodontitis.

b Model 2: Binominal logistic regression model adjusted for age, sex, education, smoking, toothbrushing frequency, low HDL-C, and hs-CRP.

### Associations between ANRIL genotype and obesity under the recessive model in the total sample, and stratified by periodontitis subgroup

In a model with obesity as the outcome variable and rs1537373 as the explanatory variable, the GG genotype was associated with higher odds for obesity in the stratified stage II (OR = 1.43, 95% CI = 1.05–1.93, *p* = 0.022) and stage III–IV (OR = 2.59, 95% CI = 1.51–4.44, *p* = 0.001) subgroups (Supplementary Table S1).

### Latent class analysis

Detailed information about this statistical analysis including diagram describing latent class model (Supplementary Figure S1), class-specific probabilities (95% CI) from three-class model (Supplementary Table S2), profile plot illustrating three classes (Supplementary Figure S2), class enumeration (Supplementary Table S3), unadjusted (Supplementary Table S4) and adjusted (Supplementary Table S5) logistic regression results for *ANRIL* genotype by latent class can be found in Supplementary Material. Three latent classes were identified. Class 3 ‘periodontitis, metabolic disbalance and low-grade inflammation’ was characterised by the high probability of periodontitis and the highest probability of metabolic disbalance, and low-grade inflammation (Supplementary Table S2). Adjusted odds of having *ANRIL* GG genotype was higher for participants in class 3 (OR = 1.63, 95% CI = 1.09–2.41, *p* = 0.015) (Supplementary Table S5).

## Discussion

This population-based study was exploratory in nature and aimed to investigate associations of genotype rs1537373 T>G of the *ANRIL* gene with periodontitis, as well as multiplicative and additive interactions between unmodifiable and modifiable risk factors. Firstly, we found no significant association between *ANRIL* genotype and periodontitis when interactions were not considered. Secondly, there were both multiplicative and additive interactions between *ANRIL* polymorphism and obesity on periodontitis stage III–IV, where the simultaneous presence of the GG genotype and obesity resulted in 2.5-fold higher odds of periodontitis. Sixty per cent of this association was explained by the joint effect of *ANRIL* risk genotype and obesity. Stratified analysis showed that participants with obesity who carried the GG genotype had 1.5- and 2.0-fold higher odds of having periodontitis stage II and III–IV, respectively, compared to non-obese participants with the GT/TT genotype. In addition to our main findings, we noticed that higher proportions of individuals with hypertriglyceridemia and family risk for CHD were GG carriers. GG genotype was associated with increased risk for obesity only in participants with periodontitis stage II and III–IV. Several aspects of our results are novel; however, the exploratory nature of this study indicates that the findings are preliminary and should be interpreted in light of its methodological limitations.

### Study limitations

This study has three main limitations: a small sample size in the subgroups, especially GG carriers with periodontitis stage III–IV and obesity (*n* = 43), the lack of replication, and that we tested only one polymorphism which belongs to a large haplotype block. A small sample size in the subgroups could result in overestimates of effect size and low reproducibility of results. The risk of Type I error is particularly relevant in exploratory analyses, where the primary aim is hypothesis generation rather than confirmation. In the interaction analyses conducted in this study, multiple subgroups were tested to explore potential associations. While this approach can provide valuable insights, it increases the risk of Type I error, where a statistically significant result may occur by chance rather than reflecting a true association. On the other hand, applying corrections for multiple comparisons can reduce statistical power, potentially obscuring true interactions. Therefore, findings from the interaction analyses should be interpreted with caution. Nonetheless, the subgroup analyses that yielded statistically significant results were both theoretically and clinically meaningful. To support our findings, we used an alternative LCA approach (see Supplementary Material online), which does not require stratification. Generally, the results based on this alternative statistical analysis supported our main findings. The primary role of LCA in this study was to uncover patterns and classify participants into distinct subgroups that may not be apparent through traditional methods. As an exploratory method, LCA was applied to provide preliminary insights into the underlying heterogeneity within the dataset. We confirmed a link of the studied *ANRIL* variant with the algorithm-based identified subgroup characterised by a high probability of periodontitis, metabolic disbalance including obesity, and low-grade inflammation. LCA contributed to a deeper understanding of the complex relationships between variables, aligning with the study’s exploratory objectives. While LCA supports the main findings, some methodological limitations may introduce bias and affect the reproducibility of the findings. LCA is a data-driven approach; thus, the latent classes identified in this study may not generalise to other populations or datasets. Additional limitations include subjective decisions, such as determining the number of classes and their interpretability. The genetic base of periodontitis is complex; we had tested only one *ANRIL* polymorphism rs1537373 which belongs to a large haplotype block. Other polymorphisms (e.g. rs10757278 (strong link to CAD and myocardial infarction), rs1333048 and rs1333049 (increased risk of CAD and periodontitis), and their combinations (e.g. assessed as risk scores) might represent additional influences on the main finding. A more comprehensive understanding of the genetic contributions to the observed interactions will require the inclusion of additional polymorphisms within this block in future studies. Another limitation is that this study is cross-sectional, thus observational in nature, and the interactions we found might still be confounded by other factors. However, we adjusted for multiple confounders in our analysis.

One additional issue is that, although individuals with Stage I periodontitis are at an earlier stage of the disease, they may still be at risk of progression. Including Stage I patients in the control group could, therefore, confound the results. Nevertheless, since the participants were over 40 years old, we suggest that their risk of progression is likely low. As the present study is cross-sectional, we cannot determine the direction of associations between periodontitis, obesity, and *ANRIL* genotype. Thus, an alternative hypothesis suggesting that the joint effect of genetic risk and periodontitis can contribute to more weight gain and higher cardiovascular risk may be considered. In fact, when we constructed a model with obesity as the response variable and rs1537373 as the explanatory variable, the associations between rs1537373 and obesity were modified by periodontitis. When the total sample was stratified by periodontal status, rs1537373 was associated with obesity in stage II and stage III–IV subsamples. Validated questionnaires were used; however, some level of measurement error due to self-report is possible. Self-reported data are prone to limitations due to self-report bias, including social desirability and recall bias, which may lead to under-reporting or misreporting of such covariates as smoking, physical activity, oral hygiene, and medication use. Unfortunately, we do not have information about relationships between participants in our cohort; thus, kinship among the study participants and its confounding effect could not be completely ruled out, although the chance is minimal due to the random selection of participants. The results may be different in populations with other ethnic backgrounds. For instance, the minor allele frequency of rs1537373 is reported to be different in European populations (approx. 0.50) compared with African populations (approx. 0.89), as well as the patterns of linkage disequilibrium between rs1537373 and other SNPs in a risk haplotype. Lastly, based on our study design, the functional implications of rs1537373 are speculative. We have no available data on the binding of nuclear factors to or expression of *ANRIL* and nearby *CDKN2A/B* genes, or on the concentration of their transcripts in the peripheral blood and/or locally in periodontal tissues. For this reason, no mechanistic explanation for the role of rs1537373 in the pathogenesis of periodontitis could be provided in this study. rs1537373 resides in a non-coding region and is co-inherited with numerous candidate regulatory variants, making it challenging to determine its specific functions. The collection of functional data, such as expression quantitative trait locus (eQTL) or methylation quantitative trait locus (meQTL) analyses, was not feasible in this study due to logistical constraints, limited resources, and the complexity of the biological context. Functional investigations could be incorporated into future large-scale replication studies, as rs1537373 has previously been identified as a significant cis-eQTL for the gene *CDKN2B* [28].

### Study strengths

The current study is the first to evaluate rs1537373, periodontitis, and obesity simultaneously in a population-based survey of both women and men, and it includes a larger sample size than previous studies examining the gene-obesity interaction [11, 12]. The Tromsø7, 2015–2016 recruited participants from the general population with a wide age distribution and had relatively high attendance. The sample comprised a homogenous, mainly Norwegian population, thus confounding by ethnicity is minimal. Further, we objectively assessed periodontitis and obesity. Trained personnel performed the data collection using standardised protocols. A full-mouth dental examination was performed. We used stages based on bone loss to classify the periodontitis groups. The bone loss used to define stages is an irreversible process and therefore more suitable for studying the genetic risk [50]. Bone loss as a measure of periodontal disease progression was also chosen as the most convenient outcome in a previous longitudinal study [11].

Although common genetic pathways have been proposed, they have only been addressed in a few epidemiologic studies [10–12]. For example, beta-3-adrenergic receptor polymorphism is known to be associated with BMI and T2DM [51, 52], and in a cross-sectional study by Yoshihara et al. [12], the same polymorphism was associated with periodontal parameters, including clinical attachment loss and PD in postmenopausal women with a BMI ≥25 kg/m^2^. Interestingly, the association was stronger when the sample was restricted to women with a BMI ≥30 kg/m^2^. Moreover, the interaction between obesity and beta‐3 adrenergic receptor genotype on periodontal progression was confirmed in a subsequent longitudinal study [10]. Furthermore, a longitudinal study by Wilkins et al. [11] reported that men aged 29–64 years with obesity and specific *IL-1* genotypes were at higher risk for periodontal disease progression, based on alveolar bone loss and tooth loss during the study. Obesity intervention studies are heterogeneous and show conflicting results on the effect on periodontal outcomes [53, 54]. Our results add to a growing body of evidence that differences in genetic disposition to obesity and periodontitis may contribute to this inconsistency.

The *ANRIL* gene transcript is a long, non-coding RNA (lncRNA), which is a large class of RNA molecules that do not encode proteins [55]. Enhancer sequences at the *ANRIL* locus may influence genes within the locus and at a distance, contributing to altered genome activity patterns [43]. *ANRIL* was shown to regulate inflammatory responses mediated by different pathways, such as NF‑κB and the type I interferon signalling pathways [56].

One interesting observation in the present study is that the GG genotype of *ANRIL* rs1537373 was correlated with established risk factors for CVD [57], i.e. increased family risk for CHD and hypertriglyceridemia. This is in line with studies reporting associations between *ANRIL* rs1537373 and CHD [24, 58], but not with studies reporting the association between rs1537373 *ANRIL* and low-density lipoprotein cholesterol (LDL-C) levels [22, 23]. Overexpression of *ANRIL* may promote cholesterol efflux through binding to DNA (cytosine-5)-methyltransferase 1, enhancing the methylation, and inhibiting expression of the A Disintegrin and Metalloproteinase 10 [59].

The periodontitis subgroups can be rather heterogenous in terms of disease extent, as stage is defined by the most severely affected tooth. Also, disease activity can differ within stages, where 7% of participants with stage II bone loss and 1% of participants with stage III–IV bone loss were stable periodontitis cases with no PD >3 mm [39]. In the present study, deep periodontal pockets ≥ 6mm were detected in 14% of the stage II group, and 45% of the stage III–IV group, meaning disease activity increases with severity of bone loss (data not shown). By using bone loss to define the stages of periodontitis, individuals with either past or present periodontitis are detected and grouped. Classifications that incorporate severity of current as well as previously experienced conditions are more relevant when addressing genetic risks. Periodontitis is a chronic inflammatory disease, in which multiple causal components interact and play their etiological roles simultaneously. Therefore, we examined the interaction between genetic risk and other risk factors, including obesity on periodontitis, and assessed the magnitude of interaction on both multiplicative (statistical interaction) and additive (biological interaction) scales. We demonstrated that genetic risk factors might not be significant when they were not considered jointly with environmental risk factors such as obesity in statistical models. Interestingly, we found that the coexistence of the GG genotype of rs1537373 and obesity was additively associated with periodontitis, meaning that their joint effect was higher than the sum of the individual effects. This finding is directly relevant to public health and clinical practice, because it suggests that normalisation of weight and adherence to a healthy lifestyle may be more beneficial for periodontal health among those with genetic risks than those without such risks. We have pointed out previously in the discussion that *ANRIL* is not translated into a protein but is categorised as a lncRNA. LncRNA expression is often tissue- and condition-specific, thus LncRNAs might be considered as potential biomarkers and interesting therapeutic targets [60].

## Conclusions

In conclusion, this research serves as a preliminary investigation into the complex pattern of interaction between *ANRIL* (rs1537373) T>G and obesity on periodontitis. The results suggest that a combination of rs1537373-G and obesity may be associated with advanced periodontitis. As the genetic factors are not yet modifiable in the clinical setting, public health interventions to control the rising prevalence of obesity have the potential to reduce periodontitis risk in genetically predisposed individuals. As we observed higher proportions of individuals with hypertriglyceridemia, a family history of CHD, and obesity among GG carriers, the potential contribution of this SNP to CVD risk is suggested and warrants further investigation. These results appear to support the hypothesis that the association between obesity and periodontitis might be influenced by certain genotypes. This hypothesis should be verified in future studies that incorporate gene expression analysis, epigenetic modifications, and protein-level studies, while also involving larger populations, including diverse ethnic groups. We believe that our results may be interesting for other research groups that plan new studies involving the analysis of polymorphisms in the *ANRIL* gene. In future studies, alongside investigating other relevant *ANRIL* polymorphisms (e.g. rs10757278, rs1333042, rs1333048, rs1333049, rs2383206-8, rs10757274, rs2891168, rs10811661, rs10811656, rs4977574, and rs496892), it will be important to analyse the expression of both circular and linear *ANRIL*, as well as *CDKN2A* and *CDKN2B*. In addition, examining the expression and concentration of biomarkers commonly studied in the context of obesity, metabolic syndrome, and related inflammatory conditions (e.g. IL-1β, IL-6, IL-8, IL-10, IFN-γ, TNF-α, leptin, adiponectin and their receptors, melanocortin 4 receptor, estrogen and its metabolites, markers of lipid peroxidation, and insulin) may provide valuable insights in replication studies. Furthermore, markers of bone destruction in periodontitis are biologically plausible and should be considered. Finally, if carrying two copies of the rs1537373 G allele is a causal risk factor, we should be able to detect differential treatment responses in individuals carrying these alleles. Future human studies should aim to determine whether individuals respond differently to periodontitis and/or diabetes treatments based on the presence of two copies of the rs1537373 G allele. Of particular interest is whether treatment responses to Glucagon-Like Peptide-1 (*GLP-1*) receptor agonists, especially when combined with diet and exercise, differ depending on the underlying presence of chronic periodontitis.

## Supplementary Material



## Data Availability

This research uses data from the Tromsø7, 2015–2016. Data are available upon application to the Tromsø Study, but restrictions apply to the availability of these data, which were used under license for the current study, and so the dataset generated and analysed during the current study is not publicly available. The data can be made available from the Tromsø Study upon application to the Tromsø Study Data and Publication Committee. The legal restriction on data availability has been set by the Tromsø Study Data and Publication Committee to control data sharing, including the publication of data sets with the potential of reverse identification of de-identified sensitive participant information. The links to the main questionnaires used in different surveys of the Tromsø Study can be found on the Tromsø Study’s website (https://uit.no/research/tromsostudy). A detailed information on the variables collected can be found in Helsedata (https://helsedata.no/en/variables/?datakilde=K_TR&page=search).
