# Supplementary material for: An explorative study on the interaction of cyclin-dependent kinase inhibitor 2B antisense RNA 1 (CDKN2B-AS1/ANRIL) gene polymorphism with obesity on periodontitis among Norwegian adults
Source: Acta Odontol Scand. 2025 Aug 14;84:44368. doi: 10.2340/aos.v84.44368 (PMC12372528; doi:10.2340/aos.v84.44368)
Supplement: Supplementary file 1 [file AOS-84-44368-s1.pdf]

Supplementary material has been published as submitted. It has not been copyedited or typeset by Acta Odontologica Scandinavica.

An explorative study on the interaction of cyclin-dependent kinase inhibitor 2B antisense RNA 1 (*CDKN2B-AS1/ANRIL*) gene polymorphism with obesity on periodontitis among Norwegian adults

Natalia Petrenya, Laila A. Hopstock, Elin Hadler-Olsen, Farah Asa'ad, Lena Larsson,

Svetlana N. Zykova, Gro Eirin Holde, Alexandre R Vieira, Birgitta Jönsson

Supplementary information files

List of Tables and Figures

Supplementary Table S1. Associations between *ANRIL* genotype and obesity under a recessive model in the total sample and stratified by periodontitis subgroup.

Supplementary Figure S1. Diagram describing latent class model.

Supplementary Table S2. Class-specific probabilities (95% CI) from three-class model (N=3508).

Supplementary Figure S2. Profile plot illustrating three classes identified from periodontal and metabolic characteristics.

Supplementary Table S3. Akaike's information criterion (AIC) and Bayesian information criterion (BIC) for latent class analysis.

Supplementary Table S4. Unadjusted logistic regression results for *ANRIL* genotype by latent class.

Supplementary Table S5. Logistic regression results for covariates by latent class.

Supplementary Table S1. Associations between *ANRIL* genotype and obesity under a recessive model in the total sample and stratified by periodontitis subgroup.

	Obesity vs. no obesity<br>Model 1[a]<br>OR (95% CI)	p	Model 2[b]<br>OR (95% CI)	p
Total sample				
Genotype				
GT/TT	1.00		1.00	
GG	**1.24 (1.04, 1.49)**	**0.019**	**1.27 (1.05, 1.55)**	**0.016**<br>(p interaction genotype*periodontitis=**0.008)**
Stratified by three periodontitis groups				
**No periodontitis/stage I**				
Genotype				
GT/TT	1.00		1.00	
GG	1.01 (0.74, 1.36)	0.961	0.97 (0.69, 1.35)	0.841
**Stage II**				
Genotype				
GT/TT	1.00		1.00	
GG	1.21 (0.93, 1.59)	0.160	**1.43 (1.05, 1.93)**	**0.022**
**Stage III-IV**				
Genotype				
GT/TT	1.00		1.00	
GG	**2.30 (1.45, 3.66)**	**<0.001**	**2.59 (1.51, 4.44)**	**0.001**

[a]Model 1: Unadjusted logistic regression model between *ANRIL* genotype and obesity.
[b]Model 2: Logistic regression model adjusted for age, sex, education, smoking, toothbrushing frequency, low HDL-C, and hs-CRP.

Abbreviations: OR, odds ratio; CI, confidence interval; HDL-C, high-density lipoprotein cholesterol; hs-CRP, high-sensitivity C-reactive protein. Bold values denote statistical significance at the p<0.05 level.

Latent class analysis

Latent class analysis (Supplementary Fig. S1) was performed subsequently after main analysis because we wanted to confirm our findings by using an alternative statistical approach and to address methodological challenges that arise in the "traditional" subgroup analysis, including possible Type I error and low statistical power. Participants with missing indicator variables were excluded (n=46).

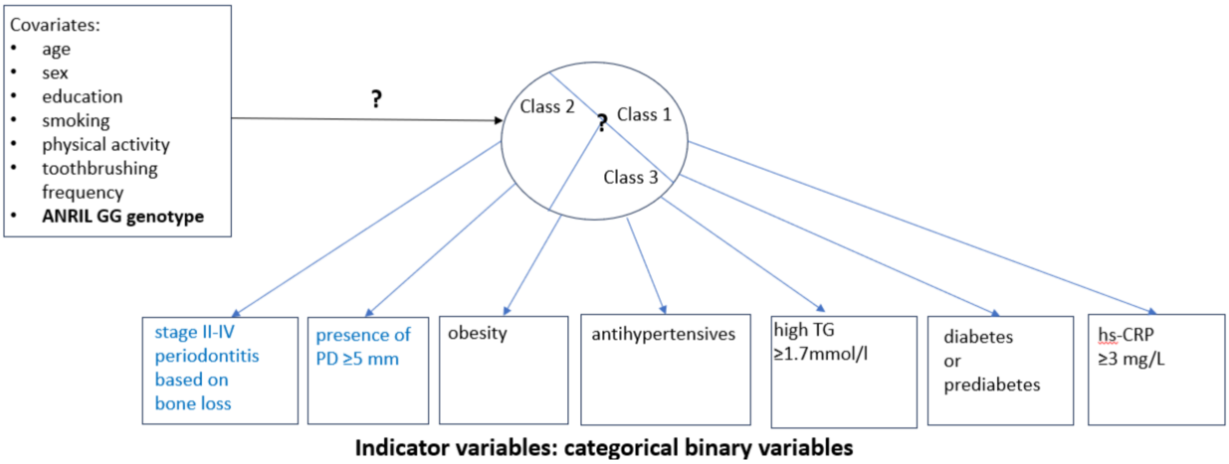

Supplementary Figure S1. Diagram describing latent class model.

Definitions: Stage II-IV based on radiographic bone loss (2018 AAP/EFP classification), obesity=BMI ≥30 kg/m^2; antihypertensives=current use by answering yes to the question "Do you use antihypertensive medication?" and/or use of antihypertensives C02, C03, C07, C08, and/or C09 from a written list of brand names of regularly used medications coded by the anatomic therapeutic and chemical (ATC) classification system; prediabetes= HbA1c ≥5.7%; diabetes= self-reported current diabetes and/or current use of diabetes medication and/or HbA1c ≥6.5%.

Abbreviations: PD, pocket depth; TG, triglycerides; hs-CRP, high sensitivity C-reactive protein; BMI, body mass index; HbA1c, glycated hemoglobin.

Hypothesis

We hypothesized that we could identify qualitatively different subgroups (classes) based on periodontal and metabolic characteristics. We suggested that class membership could depend on *ANRIL* GG genotype (Supplementary Fig. S1).

Latent classes

Participants were assigned to the class for which they had the highest posterior probability (Supplementary Table S2 and Supplementary Fig. S2). Latent class analysis identified three distinct and meaningful subgroups of participants with different periodontal and metabolic characteristics.

Class 1 "healthy" was characterised by a generally low probability of periodontitis and a low probability of metabolic disbalance.

Class 2 "periodontitis and prediabetes/diabetes" was characterised by the highest probability of periodontitis and high probability of prediabetes or diabetes.

Class 3 "periodontitis, metabolic disbalance and low-grade inflammation" was characterised by the high probability of periodontitis and the highest probability of metabolic disbalance, and low-grade inflammation.

Supplementary Table S2. Class-specific probabilities (95% CI) from three-class model (N=3508).

	Probabilities (95% CI)
class 1 "healthy" n=1570 (44.7%)	
periodontitis stage II-IV	0.26 (0.19, 0.35)
PD ≥ 5 mm	0.15 (0.11, 0.20)
triglycerides ≥ 1.7 mmol/l	0.20 (0.17, 0.23)
obesity	0.12 (0.09, 0.15)
hs-CRP ≥3 mg/l	0.06 (0.04, 0.08)
use of antihypertesives	0.08 (0.06, 0.11)
prediabetes or diabetes	0.24 (0.20, 0.28)
class 2 "periodontitis and prediabetes/diabetes" n=1011 (28.8%)	
periodontitis stage II-IV	0.95 (0.67, 0.99)
PD ≥5 mm	0.56 (0.48, 0.64)
triglycerides ≥1.7 mmol/l	0.19 (0.14, 0.24)
obesity	0.05 (0.02, 0.17)
hs-CRP ≥ 3 mg/l	0.10 (0.07, 0.14)
use of antihypertesives	0.26 (0.21, 0.31)
prediabetes or diabetes	0.49 (0.43, 0.56)
class 3 "periodontitis, metabolic disbalance and low-grade inflammation" n=927 (26.4%)	
periodontitis stage II-IV	0.67 (0.62, 0.72)
PD ≥5 mm	0.46 (0.41, 0.50)
triglycerides ≥ 1.7 mmol/l	0.52 (0.47, 0.57)
obesity	0.61 (0.52, 0.69)
hs-CRP≥ 3 mg/l	0.32 (0.28, 0.37)
use of antihypertesives	0.48 (0.43, 0.53)
prediabetes or diabetes	0.65 (0.60, 0.70)

Abbreviations: CI: confidence interval; hs-CRP: high sensitivity C-reactive protein

Supplementary Figure S2 shows profile plot illustrating three identified classes.

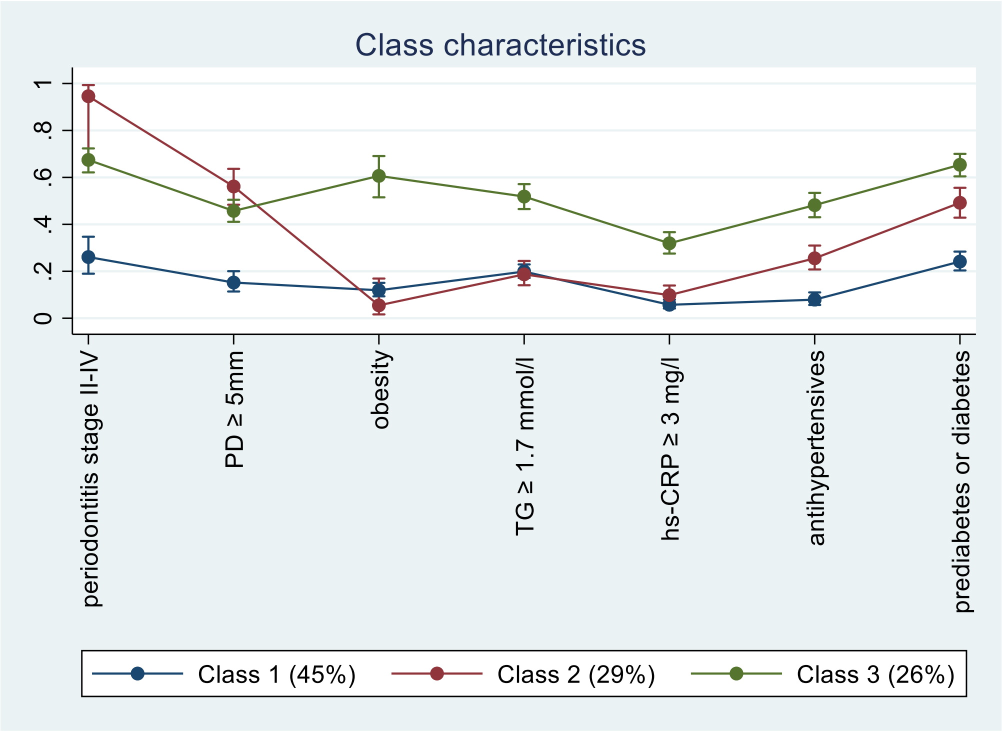

Supplementary Figure S2. Profile plot illustrating three classes identified from periodontal and metabolic characteristics. The horizontal axis shows 7 characteristics. The vertical axis shows the probabilities.

Class enumeration

Three-class solution model was chosen based on the lowest AIC and BIC and interpretability of classes (Supplementary Table S3). Entropy=0.7.

Supplementary Table S3. Akaike's information criterion (AIC) and Bayesian information criterion (BIC) for latent class analysis.

Model	AIC	BIC
Model with one class	29217.54	29260.68
Model with two classes	28487.96	28580.41
**Model with three classes**	**28297.64**	**28439.38**

Best-fitting model is indicated in bold.

Results

Multinominal logistic regression predicting class membership with Class 1 set as the reference indicated that the unadjusted (Supplementary Table S4) and adjusted (Supplementary Table S5) odds of having *ANRIL* GG genotype was higher for participants in class 3 "periodontitis, metabolic disbalance, and low-grade inflammation" than for participants in class 1.

Supplementary Table S4. Unadjusted logistic regression results for *ANRIL* genotype by latent class.

	Beta coefficient (95% CI)	Odds Ratio (95% CI)	p
**Class 1**	(base outcome)		
**Class 2**			
ANRIL genotype GG	-0.10 (-0.42, 0.23)	1.10 (0.66, 1.26)	0.560
**Class 3**			
ANRIL genotype GG	**0.35 (0.09, 0.61)**	**1.42 (1.09, 1.84)**	**0.008**

Bold values denote statistical significance at the $p<0.05$ level.

Supplementary Table S5. Logistic regression results for covariates by latent class.

	Beta coefficient (95% CI)	Odds Ratio (95% CI)	p
**Class 1**	(base outcome)		
**Class 2**			
Age	**0.21 (0.19, 0.24)**	**1.23 (1.21, 1.27)**	**<0.001**
Male sex	0.36 (-0.02, 0.75)	1.43 (0.98, 2.11)	0.063
Education			
primary	1.00	1.00	
secondary	-0.52 (-1.07, 0.04)	0.59 (0.34, 1.04)	0.068
tertiary	**-0.82 (-1.35, -0.29)**	**0.44 (0.26, 0.75)**	**0.003**
Smoking pack-years[a]	**0.12 (0.09, 0.14)**	**1.13 (1.09, 1.15)**	**<0.001**
Physical activity			
sedentary	1.00	1.00	
light	0.59 (-0.05, 1.23)	1.80 (0.95, 3.42)	0.069
moderate-to-vigorous	0.19 (-0.53, 0.90)	1.20 (0.59, 2.46)	0.609
Toothbrushing frequency < twice a day	0.03 (-0.47, 0.53)	1.03 (0.63, 1.70)	0.912
ANRIL genotype GG	0.01 (-0.47, 0.48)	1.01 (0.63, 1.62)	0.975
**Class 3**			
Age	**0.14 (0.12, 0.17)**	**1.15 (1.13, 1.18)**	**<0.001**
Male sex	**0.74 (0.39, 1.10)**	**2.09 (1.48, 3.00)**	**<0.001**
Education			
primary	1.00	1.00	
secondary	**-0.58 (-1.07, -0.10)**	**0.56 (0.34, 0.90)**	**0.019**
tertiary	**-1.46 (-1.93, -0.98)**	**0.23 (0.14, 0.37)**	**<0.001**
Smoking pack-years[a]	**0.10 (0.07, 0.12)**	**1.11 (1.07, 1.13)**	**<0.001**
Physical activity			
sedentary	1.00	1.00	
light	**-0.84 (-1.32, -0.36)**	**0.43 (0.27, 0.70)**	**0.001**
moderate-to-vigorous	**-1.65 (-2.23, -1.07)**	**0.19 (0.11, 0.34)**	**<0.001**
Toothbrushing frequency < twice a day	**0.82 (0.41, 1.23)**	**2.27 (1.51, 3.42)**	**<0.001**
ANRIL genotype GG	**0.49 (0.09, 0.88)**	**1.63 (1.09, 2.41)**	**0.015**

[a]Pack–years=the total number of years a person smoked * (number of cigarettes smoked per day cigarettes/20). Bold values denote statistical significance at the $p<0.05$ level.
